# Neural field theory of adaptive effects on auditory evoked responses and mismatch negativity in multifrequency stimulus sequences

**DOI:** 10.3389/fnhum.2023.1282924

**Published:** 2024-01-03

**Authors:** Tahereh Babaie-Janvier, Natasha C. Gabay, Alexander McInnes, Peter A. Robinson

**Affiliations:** ^1^School of Physics, The University of Sydney, Sydney, NSW, Australia; ^2^Center of Excellence for Integrative Brain Function, The University of Sydney, Sydney, NSW, Australia

**Keywords:** evoked responses, mismatch negativity, neural field theory, adaptation, oddball paradigm, stimulus discriminability

## Abstract

Physiologically based neural field theory (NFT) of the corticothalamic system, including adaptation, is used to calculate the responses evoked by trains of auditory stimuli that differ in frequency. In oddball paradigms, fully distinguishable frequencies lead to different standard (common stimulus) and deviant (rare stimulus) responses; the signal obtained by subtracting the standard response from the deviant is termed the mismatch negativity (MMN). In this analysis, deviant responses are found to correspond to unadapted cortex, whereas the part of auditory cortex that processes the standard stimuli adapts over several stimulus presentations until the final standard response form is achieved. No higher-order memory processes are invoked. In multifrequency experiments, the deviant response approaches the standard one as the deviant frequency approaches that of the standard and analytic criteria for this effect to be obtained. It is shown that these criteria can also be used to understand adaptation in random tone sequences. A method of probing MMNs and adaptation in random tone sequences is suggested to makes more use of such data.

## 1 Introduction

**Figure 1:**
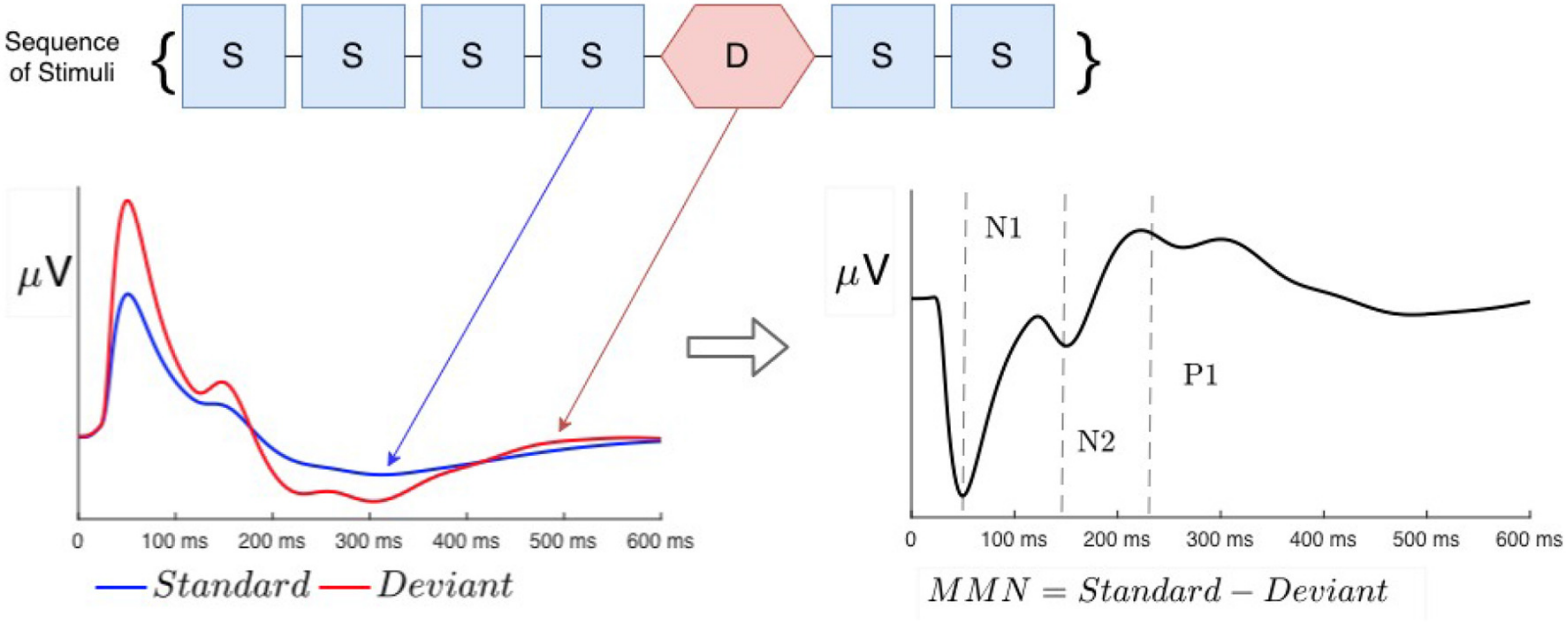
Schematic examples of typical standard (blue) and deviant (red) evoked responses to auditory stimuli in an auditory oddball experiment. Traditional phenomenological “components” (peaks and troughs) are labeled N1, N2, and P3. Note that negative signals correspond to the upward direction in these plots, in accord with convention.

Neural processing of sensory information in normal and abnormal states is commonly investigated using evoked responses (ERs) to impulsive stimuli, measured via non-invasive methods of electroencephalography (EEG) or magnetoencephalography (MEG) (Näätänen and Alho, 1997; Tervaniemi et al., 1997; Luck and Kappenman, 2011; Niedermeyer and Lopes da Silva, 2011; Luck, 2014). A so-called oddball paradigm is widely used to analyze the effects evoked by any violation of regularity—e.g., changes in frequency, location, duration, or intensity (Näätänen, 2003; Luck and Kappenman, 2011; Luck, 2014), in which a train of so-called *standard* (S) stimuli is interrupted by rarer stimuli, termed *deviant* (D), as seen in Figure 1. Such irregularities elicit very different responses when the two types of stimuli are fully discriminable, whereas marginally discriminable stimuli give an intermediate response (Sams et al., 1985; Näätänen, 2003; Garrido et al., 2009a, 2013). Throughout this article, we denote stimuli with calligraphic font to distinguish them from responses, written in italic.

Significantly, the first stimulus in a stimulus train always evokes a *D* response, whereas standard responses only emerge after a few successive presentations, approaching their limiting form *S*_∞_ over an adaptation timescale of several seconds (Näätänen, 2003; Garrido et al., 2009a, 2013; Luck and Kappenman, 2011; Luck, 2014). Involvement of adaptation is also inferred because a pause in stimulation causes *S* responses to relax to the *D* form over a few seconds (Cowan, 1984; Winkler et al., 1993; Loveless et al., 1996; Näätänen, 2003). Similarly, when two D stimuli occur consecutively or an S follows two Ds, both *D* and *S* responses differ from their prototypical forms (Sams et al., 1984). This implies that adaptation to recent stimuli is at least partly responsible for the different responses. It thus counts against interpretations that assert that the system establishes expectations of the long-term statistical properties of incoming stimuli (Näätänen et al., 1978, 1989, 1993, 2005, 2010; Tiitinen et al., 1993; Kraus et al., 1995, 1996; Tervaniemi et al., 1997; Atienza et al., 2001; Näätänen, 2003; Garrido et al., 2009b; Luck and Kappenman, 2011; Luck, 2014), although it does not rule out some contribution from such effects.

A *D* response is also evoked by other irregularities within a train of stimuli, including a repeated tone in an otherwise descending sequence where no prior tone is repeated (Näätänen et al., 1989; Tervaniemi et al., 1997; Näätänen, 2003; Garrido et al., 2009b, 2013); after a stimulus that is omitted or changed in duration or intensity (Näätänen et al., 1989, 2007; Yabe et al., 1997; Näätänen, 2003; Salisbury, 2012); or when the overall frequency range of an ensemble of random stimuli exceeds the discriminability threshold (Sams et al., 1985; Garrido et al., 2013).

ERs are most commonly phenomenologically parameterized by the timings and amplitudes of so-called *components*, which approximately correspond to peaks and troughs in the waveform (Luck and Kappenman, 2011; Luck, 2014). It is widely assumed that each component has a fixed timing (latency) and polarity (positive or negative) in normal subjects and that cognitive processes only change their amplitudes (Hillyard and Anllo-Vento, 1998; Hillyard et al., 1998). In this vein, the *S* response is often subtracted from the *D* response to compute the so-called mismatch negativity (MMN), which has been argued to be a separate component that results from top-down memory-based comparison processes in higher-order cortical areas that flag deviance from a pre-established regularity (Näätänen et al., 1978, 1989, 1993, 2005, 2010; Tiitinen et al., 1993; Kraus et al., 1995, 1996; Tervaniemi et al., 1997; Atienza et al., 2001; Näätänen, 2003; Garrido et al., 2009b; Luck and Kappenman, 2011; Luck, 2014). In the present study, we base our description of ERs on the underlying physical brain activity that they reflect, and only use component terminology as a convenient shorthand to designate timings and polarities of peaks and troughs. In this notation, N1 and N2 denote negative peaks at around 100 and 200 ms post-stimulus, and P3 denotes a positive peak at 300 ms.

An alternative to the above view is that the auditory MMN is the result of cortical adaptation to repeated S stimuli that changes the *S* response at the relevant point of the tonotopic map, whereas the point that corresponds to the D stimuli undergoes little adaptation, which mostly relaxes before the next such stimulus arrives (Atienza et al., 2001; Jääskeläinen et al., 2004). This does not preclude contributions from higher-order memory processes; however, basic biophysics, the evolution of *S* and *D* responses during long trains, the decay of their distinction during a few-second stimulation pause, and the existence of MMN in coma (during which there is arguably no higher order processing) all imply a role for adaptation (Schröger, 1998; Näätänen, 2003; Jääskeläinen et al., 2004; Sussman et al., 2014). Ruusuvirta (2021) pointed out that there are still uncertainties about the precise mechanisms of adaptation and its importance in determining ER structure, which reinforces the need to test the extent to which adaptation can even potentially account for ER structure.

Our approach to testing the potential role of adaptation is to model ERs mechanistically in terms of the response of cortical activity to incoming stimuli, focusing on the effects of slow adaptation and adding frequency dependence to our prior neural field theory (NFT; Robinson et al., 2021) to see whether they can account for the evolution of ERs from deviant to standard form when driven by multiple stimuli, and for frequency-dependent features. NFT has been extensively used to model ERs, ongoing EEG characteristics, and other phenomena (Rennie et al., 2002; Robinson et al., 2002; Kerr et al., 2008, 2009, 2011; Babaie-Janvier and Robinson, 2018, 2019, 2020; Mukta et al., 2020). In particular, Kerr et al. (2011) successfully fitted NFT impulse-response models of *S* and *D* ERs to data from cohorts of up to nearly 1,500 subjects, albeit without including adaptation. They showed that inferred prestimulus parameters for *S* and *D* responses could be significantly different from each other and from those of background EEG (van Albada et al., 2010; Kerr et al., 2011). Our recent study (Babaie-Janvier and Robinson, 2018, 2019, 2020) also showed that stimulus-driven gain changes occur as part of ERs and affect their form. Most recently, Robinson et al. (2021) incorporated adaptation into the NFT model of ERs and used it to calculate *S* and *D* responses to sequences of simple stimuli, including the development of distinct response characteristics. This provides a means by which a wide range of experimental outcomes can be reproduced using a single model, including the entire waveform, not just its peaks and troughs. Moreover, it predicts observed changes in amplitudes and timings of oscillations due to changes in corticothalamic parameters, implying that fixed-latency components do not best reflect the underlying dynamics. This quantitative approach also enables one to determine how much of the dynamics can be accounted for by adaptation and what remainder might be due to higher-order top-down memory-related stimulus-comparison processes. Using these physically based approaches, they showed that the building blocks of responses are the same damped corticothalamic oscillations that account for ongoing EEG characteristics and other phenomena.

In the present study, we use our recent NFT model (Babaie-Janvier and Robinson, 2020; Robinson et al., 2021) and generalize it to incorporate the auditory tonotopic map to allow for stimuli to overlap in their adaptive effects, instead of being assumed to be entirely distinct. This method enables us to treat responses to a train of stimuli in which frequent and infrequent tones are not fully distinguishable (Robinson et al., 2021), and to relate the response characteristics to the probability distribution of random stimuli (Garrido et al., 2013). We thus aim to explore the extent to which adaptation can account for the occurrence of standard, deviant, and intermediate responses to trains of stimuli that can differ in frequency.

The structure of the article is as follows: Section 2 provides an overview of the necessary background theory for an interdisciplinary readership, followed by extension of the NFT of ERs with adaptation to also include tonotopy, in the absence of higher-order feedbacks. In Section 3, we use the model to predict the MMN as a function of stimulus discriminability in an oddball paradigm and predict responses in random tone experiments. In each case, the results are compared with experimental outcomes in the literature. Section 4 summarizes the main findings and outlines directions for future study.

## 2 Materials and methods

This material summarizes and further develops the necessary theory for our analysis. Section 2.1 briefly summarizes the relevant background aspects of the use of physiologically based neural field theory in modeling large-scale brain activity and reviews the essential components of our specific corticothalamic model, which has previously been successfully tested against experimental results in other contexts (Rennie et al., 2002; Kerr et al., 2008, 2011; Babaie-Janvier and Robinson, 2020; Robinson et al., 2021). To avoid undue repetition, we refer the reader particularly to the study by Robinson et al. (2021) for further details of the model and its mathematical treatment in both time and frequency domains, so that new aspects can be focused on here. Section 2.2 then discusses how the tonotopic map and auditory inputs are treated and establishes criteria for significant adaptation. The connection to measured ERs is then discussed in Section 2.3.

### 2.1 NFT of corticothalamic evoked responses

**Figure 2:**
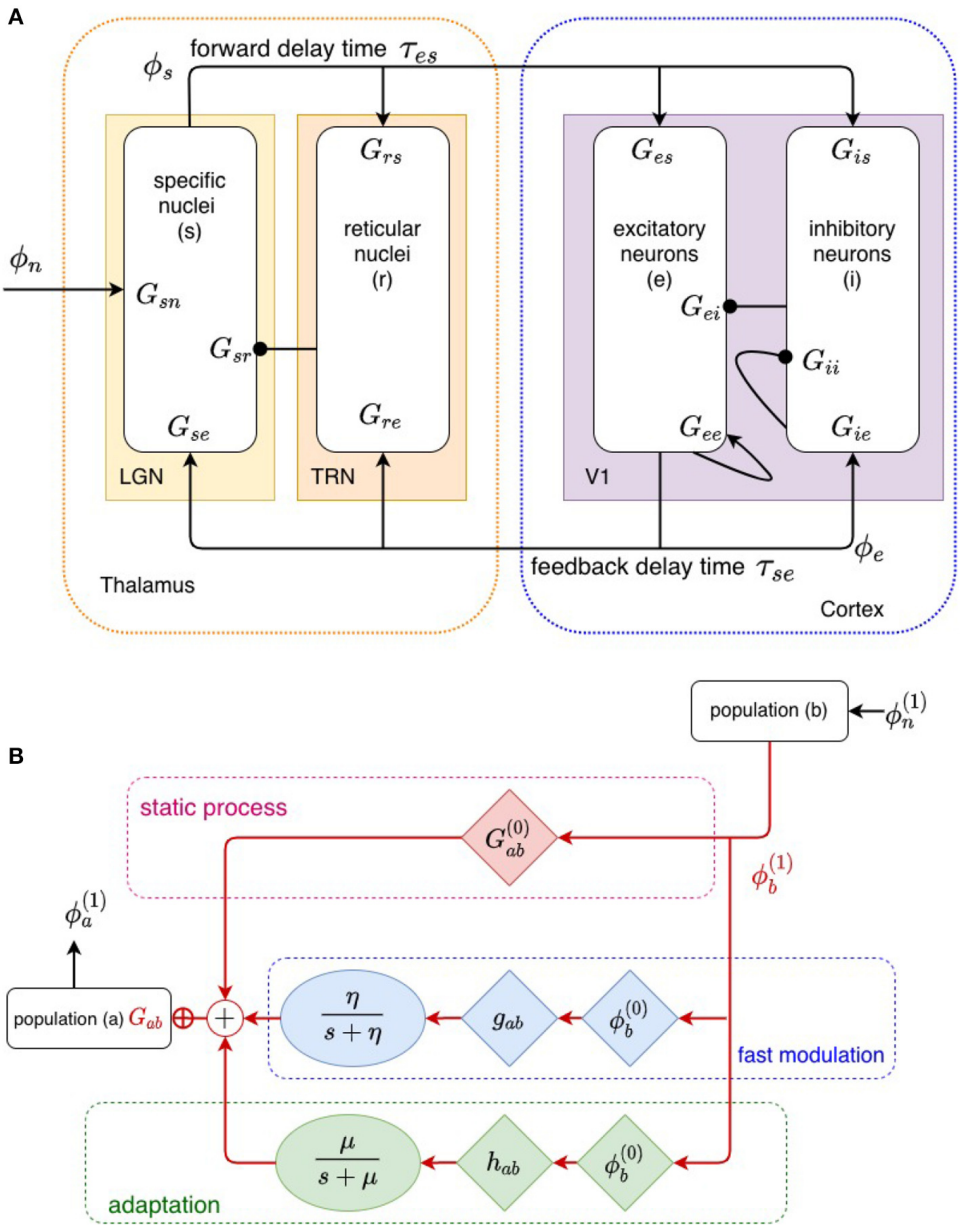
**(A)** Physiologically based corticothalamic model in which the arrows represent excitatory effects and the circles depict inhibitory ones. The populations are cortical excitatory (*e*) and inhibitory (*i*) neurons, the thalamic reticular nucleus (*r*), thalamic relay neurons (*s*) that project to the cortex, and non-corticothalamic neurons responsible for external inputs (*n*). **(B)** Schematic of propagation of neural activity to population *a* from population *b*, where both fast (through η) and slow (through μ) modulation of the neuronal gain by local feedback is given by Equation (9).

Cortical evoked responses (ERs), as measured by EEG or MEG techniques, are generated primarily by perturbations in the activity ϕ_*e*_ arriving at synapses of pyramidal excitatory cells due to dynamics in the corticothalamic system (Nunez and Cutillo, 1995). Our corticothalamic model, shown in Figure 2, incorporates the cortex and thalamus and their connectivities; each includes distinct population of neurons: cortical excitatory (*e*) and inhibitory (*i*) neurons, the thalamic reticular nucleus (TRN; *r*), thalamic relay neurons (*s*), and non-corticothalamic neurons that provide external inputs (*n*). In this study, the relevant relay nucleus is the medial geniculate nucleus (MGN), whose projections are to the primary auditory cortex (A1). The model incorporates the auditory projection system with reciprocal corticothalamic feedback projections, excitatory projections to the TRN from MGN-A1 feedforward axons and A1-MGN feedback axons, and inhibitory projections from the TRN onto MGN relay neurons.

The NFT discussed by Robinson et al. (2021) yields partial differential equations for the mean firing rates ϕ_*a*_ of neurons in the various structures mentioned in the previous paragraph, with *a* = *e, i, r, s, n*. The firing rate ϕ_*e*_ in cortical pyramidal neurons has previously been shown to be the one most closely related to EEG signals (Nunez and Cutillo, 1995; Nunez and Srinivasan, 2006), a relationship we continue to assume here.

Solution of physiologically based NFT equations of the corticothalamic model first yields spatially uniform steady states of the system, which are interpreted as characterizing the baseline of normal activity, with firing rates ϕ_*a*_ that are in accord with experiment (Robinson et al., 2002, 2004). Linear perturbations from these steady states have been shown to correspond to time dependent brain activity, leading to successful comparisons with numerous experimental phenomena, including evoked responses (Robinson et al., 1997, 2002, 2004, 2005; Rennie et al., 2002; O'Connor and Robinson, 2004; Kerr et al., 2008; van Albada et al., 2010; Roberts and Robinson, 2012; Abeysuriya et al., 2015). In this study, we consider only the large-scale global ER because it has been shown to dominate the measurable signal, as seen in Figure 12 of Mukta et al. (2020).

Application of the Laplace transform


(1) $$\begin{array}{l} L\left[f\left(t\right)\right]\left(s\right)=f\left(s\right)=\int_{0}^{\infty }f\left(t\right)e^{-st}dt, \end{array}$$

to the NFT equations yields the following equation for the activity ϕ_*a*_ at each neural population *a* in terms of activity arriving from other populations *b*:


(2) $$\begin{array}{l} {\hat{D}}_{a}\left(s\right)\left[\phi_{a}^{\left(0\right)}+\phi_{a}^{\left(1\right)}\left(s\right)\right]=\hat{L}\left(s\right)\underset{b}{\sum }G_{ab}\left[\phi_{b}^{\left(0\right)}+\phi_{b}^{\left(1\right)}\left(s\right)e^{\left(-s\tau_{ab}\right)}\right], \end{array}$$

where we retain first order perturbations (superscript 1) from the steady state (superscript 0), and


(3) $$\begin{array}{l} {\hat{D}}_{a}\left(s\right)={\left(1+s/\gamma_{a}\right)}^{2}, \end{array}$$

(4) $$\begin{array}{l} \hat{L}\left(s\right)=\alpha \beta /\left[\left(s+\alpha \right)\left(s+\beta \right)\right], \end{array}$$

where $\hat{L}\left(s\right)$ is the operator that embodies the temporal response of cell-body potentials to afferent pulse rate fields ϕ_*b*_ by encapsulating the rates β and α of the response's rise and fall, ${\hat{D}}_{a}\left(s\right)$ corresponds to a damped wave operator (Jirsa and Haken, 1996; Robinson et al., 1997) with the damping rate γ_*a*_ satisfying γ_*a*_ = *v*_*a*_/*r*_*a*_, where *r*_*a*_ and *v*_*a*_ are the characteristic range and conduction velocity of axons of type *a* (in the corticothalamic system, only the axons of excitatory cortical neurons are long enough to cause significant propagation effects on large scales; in the other populations, we assume the axonal length to be small enough that it can be neglected, whence *r*_*a*_ ≈ 0 and ${\hat{D}}_{a}\approx 1$), and the gains *G*_*ab*_, in general, are the response in neuron *a* due to unit input from neuron *b*; i.e., the number of additional pulses out for each additional pulse in.

For first-order perturbations, we can write


(5) $$\begin{array}{l} \phi_{e}^{\left(1\right)}\left(t\right)=\int_{-\infty }^{t}T_{en}\left(t-t^{\prime }\right)\phi_{n}^{\left(1\right)}\left(t^{\prime }\right)dt^{\prime }, \end{array}$$

for a purely temporal response, where the other linear perturbations have been eliminated from the equations, *T*_*en*_ is the resulting linear response function, which embodies the system linear response to a perturbation, with $T_{en}\left(t-t^{\prime }\right)=0$ for *t* < *t*′ to preserve causality. In Equation (5), $\phi_{n}^{\left(1\right)}$ is the incoming non-corticothalamic stimulus to the corticothalamic system. The form in Equation (1) can be generalized to include spatial aspects, but here we focus on the temporal domain to bring out the main aspects without undue complexity (Kerr et al., 2008). Equation (1) can be Laplace transformed to yield


(6) $$\begin{array}{l} \phi_{e}^{\left(1\right)}\left(s\right)=T_{en}\left(s\right)\phi_{n}^{\left(1\right)}\left(s\right), \end{array}$$

which expresses the transfer function as the ratio of output to input in the Laplace domain. If the input in Equation (6) is a delta function $\phi_{n}\left(t^{\prime }\right)=\delta \left(t^{\prime }-t_{0}\right)$, one finds


(7) $$\begin{array}{l} \phi_{e}^{\left(1\right)}\left(t\right)=T_{en}\left(t-t_{0}\right), \end{array}$$

whence we see that the transfer function and the ER to a delta input are one and the same. More generally, subsequent physical phenomena such as volume conduction, measurement effects, and postprocessing should be included in the overall transfer function from stimulus to measurement, but we omit discussion of these issues because they do not strongly affect the time course of large-scale ERs, which is our focus here.

The transfer function itself can be changed by the stimulus, owing to a variety of fast and slow dynamical effects that cause the gains *G*_*ab*_ to evolve in time (Koch, 1999; Rennie et al., 1999, 2000, 2002; Robinson and Roy, 2015; Babaie-Janvier and Robinson, 2019) due to current or recent activity, including plasticity, long-term potentiation/depression, adaptation, facilitation, habituation, and sensitization (Koch, 1999; Rennie et al., 2000; Robinson and Roy, 2015; Babaie-Janvier and Robinson, 2019). Rennie et al. (1999), Koch (1999), Robinson et al. (2002), and Robinson and Roy (2015) introduced a general mathematical form for gain changes that are driven by local activity and that relax toward equilibrium with a characteristic timescale that can be applied to a broad range of local feedback mechanisms in which presynaptic neuronal activity modulates neuronal gains. For moderate perturbations, it yields


(8) $$\begin{array}{l} G_{ab}\left(s\right)=G_{ab}^{\left(0\right)}+G_{ab}^{\left(1\right)}\left(s\right), \end{array}$$

(9) $$\begin{array}{l} G_{ab}^{\left(1\right)}\left(s\right)=\left[g_{ab}F\left(s\right)+h_{ab}H\left(s\right)\right]\phi_{b}^{\left(1\right)}\left(s\right), \end{array}$$

where $G_{ab}^{\left(0\right)}$ is the static gain and $G_{ab}^{\left(1\right)}$ is the gain perturbation caused by local feedback. Here, *F*(*t*) describes the temporal dynamics of fast gain modulation on timescales of up to a few hundred ms and *g*_*ab*_ is its strength, whereas *H*(*t*) is a slow adaptation process on timescales of 5 – 10 s, with *h*_*ab*_ the corresponding strength; *g*_*ab*_ and *h*_*ab*_ are assumed constant in the present study. Figure 2 depicts this modulation schematically. Gain dynamics driven by postsynaptic firing is postponed to future study, but can be treated similarly (Rennie et al., 1999; Robinson and Roy, 2015; Robinson et al., 2021). For simplicity, we use the forms


(10) $$\begin{array}{l} F\left(s\right)=\eta /\left(s+\eta \right), \end{array}$$

(11) $$\begin{array}{l} H\left(s\right)=\mu /\left(s+\mu \right). \end{array}$$

In the time domain, *F*(*t*) = *H*(*t*) = 0 for *t* < 0 to enforce causality, while the positive rate constants η and μ are the inverse timescales of the modulatory processes and the forms (10) and (11) are normalized to unit integral over time. Previous study found η = 25 s^−1^ (Rennie et al., 1999; Babaie-Janvier and Robinson, 2019), and later study set μ = 0.65 s^−1^ because of the several-second timescales over which *S* response characteristics develop and decay (Robinson et al., 2021). Substituting the dynamic form of *G*_*ab*_ from Equations (8) and (9) into Equation (2), one finds


(12) $$\begin{array}{l} {\hat{D}}_{a}\left(s\right)\phi_{a}^{\left(1\right)}\left(s\right)=\hat{L}\left(s\right)\underset{b}{\sum }\left[G_{ab}^{\left(0\right)}e^{-s\tau_{ab}}+\phi_{b}^{\left(0\right)}\left\{g_{ab}F\left(s\right)+h_{ab}H\left(s\right)\right\}\right]\phi_{b}^{\left(1\right)}\left(s\right), \end{array}$$

the right side of which expresses two types of first-order responses: the first term in the square brackets is the response that would occur without change to the steady-state gains, while the second term is the response due to stimulus-induced gain changes acting on the steady-state activity (Robinson et al., 2021).

It is straightforward to eliminate the other first-order quantities to obtain the transfer function to excitatory cortical activity from auditory signals that reach the thalamus (Babaie-Janvier and Robinson, 2019, 2020), giving


(13) $$\begin{array}{l} T_{en}\left(s\right)=\frac{\phi_{e}^{\left(1\right)}\left(s\right)}{\phi_{n}^{\left(1\right)}\left(s\right)}=\frac{\chi_{esn}\left(s\right)}{M_{c}\left(s\right)P_{t}\left(s\right)-P_{c}\left(s\right)}, \end{array}$$

which expresses the ratio of the response change $\phi_{e}^{\left(1\right)}$ to a change in the input $\phi_{n}^{\left(1\right)}$ (i.e., to a stimulus). The full analysis shows that the various terms in this equation have the specific forms (Robinson et al., 2021)


(14) $$\begin{array}{l} \chi_{ab}\left(s\right)=\hat{L}\left(s\right)\left[G_{ab}^{\left(0\right)}e^{-s\tau_{ab}}+\phi_{b}^{\left(0\right)}\left\{g_{ab}F\left(s\right)+h_{ab}H\left(s\right)\right\}\right], \end{array}$$

(15) $$\begin{array}{l} M_{c}\left(s\right)={\hat{D}}_{e}\left(1-\chi_{ei}\right)-\chi_{ee}, \end{array}$$

(16) $$\begin{array}{l} P_{t}\left(s\right)=1-\chi_{srs},, \end{array}$$

(17) $$\begin{array}{l} P_{c}\left(s\right)=\chi_{ese}+\chi_{esre}, \end{array}$$

χ_*abc*_ = χ_*ab*_ χ _*bc*_. Table 1 lists nominal values of model parameters for resting EEG (Robinson et al., 2004) and gain modulation parameters calibrated and used in previous studies (Babaie-Janvier and Robinson, 2019, 2020; Robinson et al., 2021). These values were estimated for normal adults and have been extensively used and verified in comparisons with experiments, as mentioned in Section 1.

**Table 1:** Estimated brain parameters for normal adults in the alert eyes-open state.

**Quantity**		**Value**		**Gain**		**Dynamic gain**	
γ_*e*_	Damping rate	109 s^−1^	*ab*	[_*G*_*ab*_]*i*_	[_*G*_*ab*_]*f*_	Fast	
α_*ab*_	Decay rate	80 s^−1^	*ee*	7.03	8.49	η*g*_*ee*_	−0.0046
β_*ab*_	Rise rate	320 s^−1^	*ei*	−8.11	−12.48	η*g*_*ei*_	0.0307
τ_*es*_	Forward delay	20 ms	*es*	1.77	1.23	η*g*_*es*_	0.0297
τ_*se*_	Feedback delay	60 ms	*se*	2.47	0.63	η*g*_*se*_	0.0042
			*sr*	−1.89	−2.15	η*g*_*sr*_	−0.0056
			*rs*	0.22	0.23	η*g*_*rs*_	0.0046
$\phi_{e}^{\left(0\right)}$	*e* Firing rate	16 s^−1^	*re*	1.31	1.18	η*g*_*re*_	0.0113
$\phi_{s}^{\left(0\right)}$	*s* Firing rate	16 s^−1^	*sn*	0.8	1.97	η*g*_*sn*_	0
$\phi_{r}^{\left(0\right)}$	*r* Firing rate	16 s^−1^				Adaptive	
$\phi_{n}^{\left(0\right)}$	*n* Firing rate	16 s^−1^				μ*h*_*ee*_	0.0420
						μ*h*_*ei*_	−0.1204
η	Fast gain-change rate	25 s^−1^				μ*h*_*es*_	−0.0098
						μ*h*_*se*_	−0.0535
μ	Adaptation rate	0.65 s^−1^				μ*h*_*sr*_	−0.0051
						μ*h*_*rs*_	0
						μ*h*_*re*_	0
						μ*h*_*sn*_	0.01

Adapted from Robinson et al. (2004), Kerr et al. (2008), and Robinson et al. (2021), the estimated values are a self-consistent nominal set of parameters that provide a good fit to standard and deviant responses. The gains [_*G*_*ab*_]*i*_ and [_*G*_*ab*_]*f*_ are the values initially and after 10 stimuli, respectively.

### 2.2 Stimulus profile at auditory cortex

In our previous study (Robinson et al., 2021), we assumed that S and D stimuli could be clearly distinguished via a large frequency separation and that EEG electrodes just responded to the total response without distinguishing spatial locations. In the current study, we relax the first assumption but retain the second. This requires us to examine the frequency content of the stimulus and its mapping to auditory cortex.

#### 2.2.1 Tone-burst stimulus

ER experiments typically use short tone bursts of sinusoidal waves of frequency *f*_0_ and duration τ. Such a burst can be written


(18) $$\begin{array}{c} X\left(t\right)=\sin\left(2\pi f_{0}t\right)W\left(t\right)\left[H\left(t\right)-H\left(t-\tau \right)\right], \end{array}$$

where Θ(*u*) is the step function


(19) $$\begin{array}{c} \Theta \left(u\right)=\{\begin{array}{cc} 1, & 0\leq u,\\ 0, & u<0. \end{array} \end{array}$$

**Figure 3:**
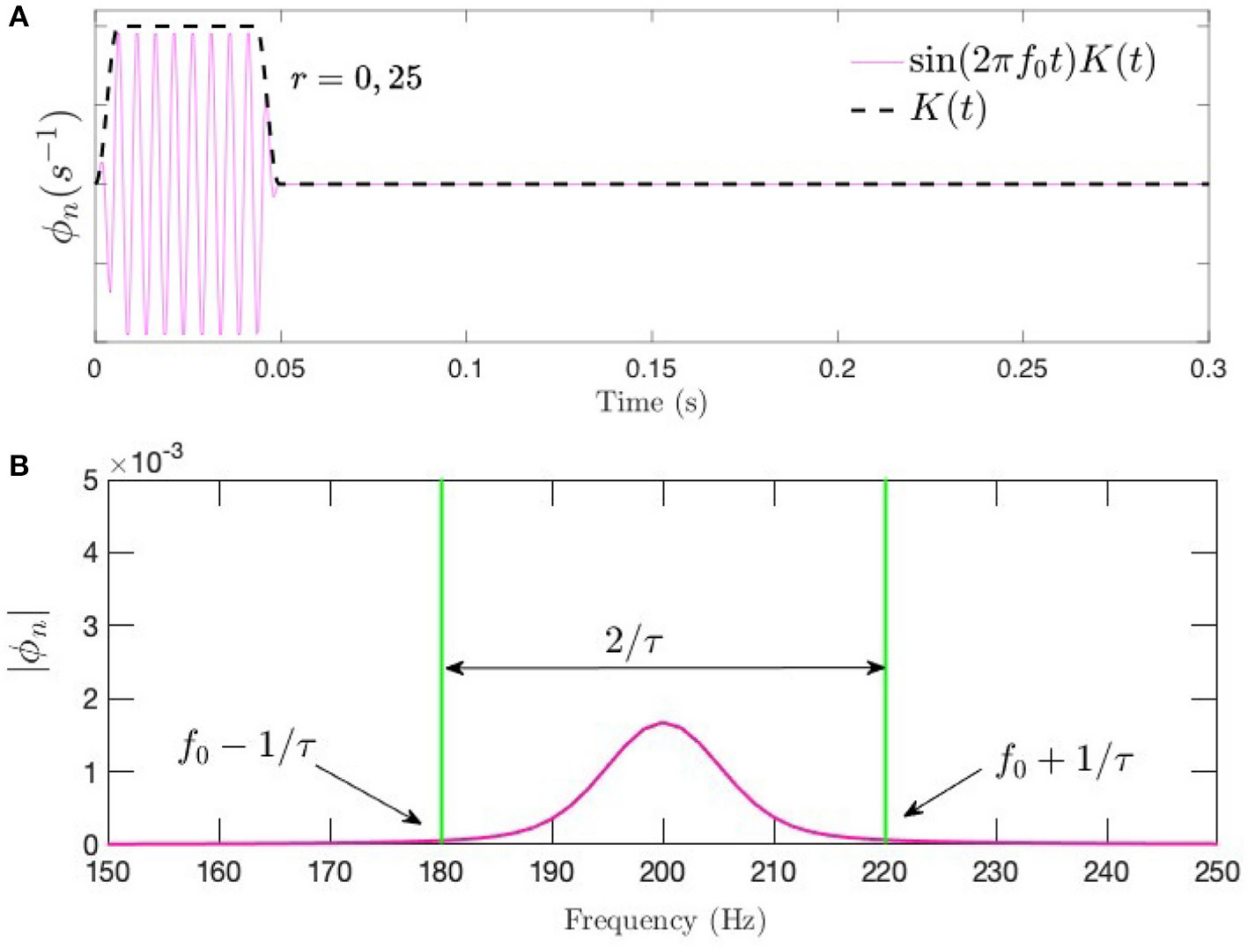
Tone burst of the form in Equation (18) at *f*_0_ = 200 Hz and τ = 50 ms with *p* = 0.2. **(A)** Time series showing the sinusoidal signal modulated by the Tukey window. **(B)** Corresponding frequency spectrum.

In Equation (18), the difference of the two step functions restricts the stimulus to the interval 0 < *t* < τ and we use the notation X to indicate that this is the externally applied stimulus, which still has to be transduced by auditory pathways before arriving at the cortex as ϕ_*n*_. The remaining factor in Equation (18) is the window function *W*(*t*) that determines the shape of the burst within the overall interval τ. To minimize the generation of side-lobes at frequencies far from *f*_0_, we use the Tukey window


(20) $$\begin{array}{c} W\left(u\right)=\{\begin{array}{cc} \sin^{2}\left[\frac{\pi t}{2p\tau }\right], & u<p\tau ;\\ 1, & \rho \tau <u<\left(1-p\right)\tau ;\\ \sin^{2}\left[\frac{\pi \left(\tau -t\right)}{2p\tau }\right], & \left(1-p\right)\tau <u; \end{array} \end{array}$$

which smooths the burst over an interval *pτ* at each end of the interval, with *p* < 0.5; we use *p* = 0.2. Fourier or Laplace transformation of Equation (18) implies that a frequency range Δ*f*_*u*_ ≈ 2/[(1 − ρ)τ] is present in a tone burst (Bracewell, 1986), which expresses the frequency–time uncertainty relation. Figure 3 shows a tone burst of *f*_0_ = 200 Hz and τ = 50 ms with *p* = 0.2, along with its Fourier spectrum.

#### 2.2.2 Transfer to the auditory cortex via the tonotopic map

When an auditory stimulus arrives at the ear, it passes via the eardrum and stapes to the cochlea, which narrows progressively with distance. Cilia near the entrance respond most strongly to low-frequency signals, while those further in respond to higher frequencies. Each group of cilia stimulates neurons that correspond to a narrow range of frequencies around its preferred one, with a firing rate that is proportional to the logarithm of the intensity (Pickles, 2013). Thus, when a frequency is present, the corresponding neurons are active with a firing rate that depends on the intensity of the signal at that frequency.

Neurons at various stages of the auditory pathway remain topographically arranged according to their optimal frequency response. This *tonotopic* organization mirrors the distribution of receptors in the cochlea, with a gradient extending between neurons that preferentially respond to high frequencies and those that respond best to low frequencies. Tonotopy is preserved via the medial geniculate nucleus (MGN) of the thalamus to the primary auditory cortex, where frequencies are approximately logarithmically spaced in a one-dimensional tonotopic map, in which each frequency *f* is mapped to a position *x*(*f*) (Talavage et al., 2004; Herdener et al., 2013; Saenz and Langers, 2014).

There is some spreading of neural projections in the pathways to the auditory cortex. This means that a pure tone of a certain frequency *f*_0_ stimulates cortical neurons across a small range of adjacent locations around *x*(*f*_0_), corresponding to a frequency range Δ*f*_nat_, which is around 0.3% of *f*_0_ for frequencies of order 1–2 kHz, which are typical in ER experiments, and about 3 Hz for frequencies below 1,000 Hz.

Any sinusoidal wave train that is cut short to a time interval of length Δ*T* to make a tone burst has an unavoidable spread in frequency Δ*f*_*u*_ ≈ 1/*T*, via the uncertainty principle. Hence, the total spread of cortical stimulation corresponds to a spread of frequencies Δ*f*, with


(21) $$\begin{array}{c} \Delta f\approx \sqrt{{\left(\Delta f_{\text{nat}}\right)}^{2}+{\left(\Delta f_{u}\right)}^{2}}. \end{array}$$

Since it is mathematically impossible to say simultaneously exactly which frequencies are present at which times during a short burst, due to the uncertainty principle, we approximate their effect on the cortex by assuming that they are all present throughout the interval of the burst. This approximation is well justified for durations of only a few tens of ms because the dynamics of the cortical response effectively integrate over the burst which appears like a delta-function in time if it is sufficiently short. Therefore, the stimulus that arrives at the primary auditory cortex can be approximated as


(22) $$\begin{array}{c} \phi_{n}\left(x,s\right)=\int_{-\infty }^{+\infty }w\left(x-x_{0}\right)S\left(f_{0},s\right)dx, \end{array}$$

where *x*_0_ = *x*(*f*_0_) and ∫*w*(*x* − *x*_0_)*dx* = 1. A suitable approximate form is


(23) $$\begin{array}{c} w\left(x-x_{0}\right)=\frac{1}{\Delta f\sqrt{2\pi }}\exp\left[-\frac{{\left(x-x_{0}\right)}^{2}}{2{\left(\Delta f\right)}^{2}}\right]. \end{array}$$

It is worth noting that the stimulus profile at the cortex can be calculated in one of two nearly equivalent ways: (i) first calculate the stimulus spectrum for given *f*_0_ and τ, giving its intrinsic width ~Δ*f*_*u*_ and then convolve the spectrum with the function that governs the spread Δ*f*_nat_ of afferents to the auditory cortex to yield the total spread on the tonotopic map, or (ii) first specify *f*_0_ and Δ*f*_*u*_ and then use the total spread from the above equation to estimate the spread on the tonotopic map directly. Here, we use the latter approach, which leads to a more straightforward implementation.

#### 2.2.3 Criteria for significant adaptive effects

Now that Δ*f* has been defined, we can now easily define criteria that characterize when significant adaptive effects due to one stimulus will affect another to produce a more *S*-like second response.

If a tone burst of central frequency *f* = *f*_0_ and duration τ occurs at *t* = *t*_0_, it causes adaptive changes within a neighborhood of *f*_0_ of width Δ*f*. These changes last a time *t*_*H*_ ≈ 5 − 10 s, so any stimulus occurring at *f*_1_ in that frequency range and *t*_1_ in that time interval will encounter a region of auditory cortex that has undergone adaptation due to the first stimulus. It is convenient to use the following two parameters when investigating adaptive effects:


(24) $$\begin{array}{c} \rho =\frac{\left|f_{0}-f_{1}\right|}{\Delta f}, \end{array}$$

(25) $$\begin{array}{c} \zeta =R\left(f_{0},\Delta f\right)t_{H}. \end{array}$$

The discriminability ρ is the ratio of the frequency separation to the spectral width Δ*f* of $\phi_{n}^{\left(1\right)}$ and is large when the two spectra stimulate quite different parts of the auditory cortex. The quantity ζ is the product of the rate *R*(*f*_0_, Δ*f*) at which stimuli arrive within Δ*f* of *f*_0_ and the adaptation timescale *t*_*H*_ and represents the mean number of stimuli that might potentially be affected by the first stimulus, or equivalently, the number of previous stimuli that might affect it. Significant adaptive effects will only occur if ρ ≲ 1 and ζ ≳ 1.

### 2.3 Measured ER

Once the stimulus $\phi_{n}^{\left(1\right)}\left(f,t\right)$ is known as a function of frequency and time on the primary auditory cortex, its local contributions to the ER, including adaptation, can be calculated using Equations (5) and (13). In the present case, we assume that the recording electrode responds to the whole stimulated cortical area, so the measured ER is obtained by integrating over all frequencies.

#### 2.3.1 ER: first stimulus

Let us consider the first ER in a sequence, so there has been no prior adaptation and the transfer function does not depend on position *x* (i.e., on frequency). The cortical response to $\phi_{n}^{\left(1\right)}\left(x,s\right)$ is


(26) $$\begin{array}{c} \phi_{e}^{\left(1\right)}\left(x,s\right)=T_{en}\left(s\right)\phi_{n}^{\left(1\right)}\left(x,s\right), \end{array}$$

(27) $$\begin{array}{c} =T_{en}\left(s\right)\int w\left(x-x^{\prime }\right)\phi_{c}^{\left(1\right)}\left(x^{\prime },s\right)dx^{\prime }, \end{array}$$

because


(28) $$\begin{array}{c} \phi_{n}^{\left(1\right)}\left(x,s\right)=\int w\left(x-x^{\prime }\right)\phi_{c}^{\left(1\right)}\left(x^{\prime },s\right)dx^{\prime }. \end{array}$$

Here, we have written $\phi_{c}^{\left(1\right)}$ for the auditory neural signal that would arrive from the cochlea if there were no spreading due to anatomical effects or the finite duration of the tone burst, while the weight function *w*(*x* − *x*′) is used to incorporate both these effects. This function has central peak and a characteristic width Δ*x* that corresponds to Δ*f*, and is normalized to satisfy


(29) $$\begin{array}{c} \int w\left(x-x^{\prime }\right)dx^{\prime }=1; \end{array}$$

We also assume that *w* is symmetric, with *w*(*x* − *x*′) = *w*(*x*′ − *x*), as in the example in Equation (23). This formulation is simpler than, but not quite as accurate as, the alternative of *w* representing only the spread due to Δ*f*_nat_ and using the actual spectral profile of the tone burst, as discussed in Section 2.2.2.

Because we assume that the electrode that detects the ER does not resolve the fine spatial scales of the tonotopic map, we must integrate the response over *x*, so we find, aside from an overall normalization,


(30) $$\begin{array}{c} \text{ER}\left(s\right)=\int \text{ER}\left(x,s\right)dx, \end{array}$$

(31) $$\begin{array}{c} \text{ER}\left(x,s\right)=\phi_{e}^{\left(1\right)}\left(x,s\right), \end{array}$$

(32) $$\begin{array}{c} =T_{en}\left(s\right)\int w\left(x-x^{\prime }\right)\phi_{c}^{\left(1\right)}\left(x^{\prime }\right)dx^{\prime }, \end{array}$$

from Equations (26) and (28). Hence, upon substituting Equation (32) into Equation (30), we obtain


(33) $$\begin{array}{c} \text{ER}\left(s\right)=\int T_{en}\left(s\right)\left[\int w\left(x-x^{\prime }\right)\phi_{c}^{\left(1\right)}\left(x^{\prime },s\right)dx^{\prime }\right]dx, \end{array}$$

(34) $$\begin{array}{c} =T_{en}\left(s\right)\int \left[\int w\left(x-x^{\prime }\right)dx\right]\phi_{c}^{\left(1\right)}\left(x^{\prime },s\right)dx^{\prime }, \end{array}$$

(35) $$\begin{array}{c} =T_{en}\left(s\right)\int \phi_{c}^{\left(1\right)}\left(x^{\prime },s\right)dx^{\prime }, \end{array}$$

from Equation (29). Hence, the ER is the response to the total integrated signal that arrives at the auditory cortex at the frequency represented by *s*.

#### 2.3.2 ER: subsequent stimuli

Because cortical stimulation from a tone burst centered at *f*_0_ is strongest at *x*_0_ = *x*(*f*_0_), this point will experience the strongest adaptation, with adaptive changes falling off with distance (i.e., with the frequency difference). As a result, we must replace *T*(*s*) by *T*(*x, s*) in Equation (33) when the next stimulus arrives, as in previous studies where *x* was ignored. Specifically, *T*(*x, s*) is calculated by inserting the instantaneous values of the *G*_*ab*_(*t*) into Equation (13), which will include long-lasting adaptive changes in general. This yields


(36) $$\begin{array}{c} \text{ER}\left(x,s\right)=T_{en}\left(x,s\right)\int w\left(x-x^{\prime }\right)\phi_{c}^{\left(1\right)}\left(x^{\prime },s\right)dx^{\prime }, \end{array}$$

in place of Equation (33). Hence, upon substituting Equation (36) into Equation (30), interchanging the order of integration, and recalling that *w* is symmetric, we find


(37) $$\begin{array}{c} \text{ER}\left(s\right)=\int \int w\left(x-x^{\prime }\right)T_{en}\left(x,s\right)\phi_{c}^{\left(1\right)}\left(x^{\prime },s\right)dx^{\prime }dx, \end{array}$$

(38) $$\begin{array}{c} =\int \left[\int w\left(x^{\prime }-x\right)T_{en}\left(x,s\right)dx\right]\phi_{c}^{\left(1\right)}\left(x^{\prime },s\right)dx^{\prime }, \end{array}$$

(39) $$\begin{array}{c} =\int T_{\text{eff}}\left(x^{\prime },s\right)\phi_{c}^{\left(1\right)}\left(x^{\prime },s\right)dx^{\prime }. \end{array}$$

Here, an effective transfer function has been defined to be


(40) $$\begin{array}{c} T_{\text{eff}}\left(x^{\prime },s\right)=\int w\left(x^{\prime }-x\right)T_{en}\left(x,s\right)dx. \end{array}$$

This implies that the effective transfer function at *x*′ is a weighted average of those at neighboring points. Hence, if the core of a stimulated region has undergone strong adaptation, its effects will be mixed with those of edge regions where adaptation is weaker, thus leading to a mixture of dominant S-like and weaker D-like features in the ER. The result (39) reproduces our previous study if *w* is approximated as being very narrow. Then, Equation (40) yields $T_{\text{eff}}\left(x^{\prime },s\right)\approx T_{en}\left(x^{\prime },s\right)$. If *w* is a delta function (which is not possible in the real system), then $T_{en}\left(x^{\prime },s\right)\approx T_{en}\left(x_{0},s\right)$, and we recover Equation (33) and our previous results if the redundant first argument is omitted. The fact that *w* always has a non-zero width implies that some mixing of characteristics will always occur.

## 3 Results

**Figure 4:**
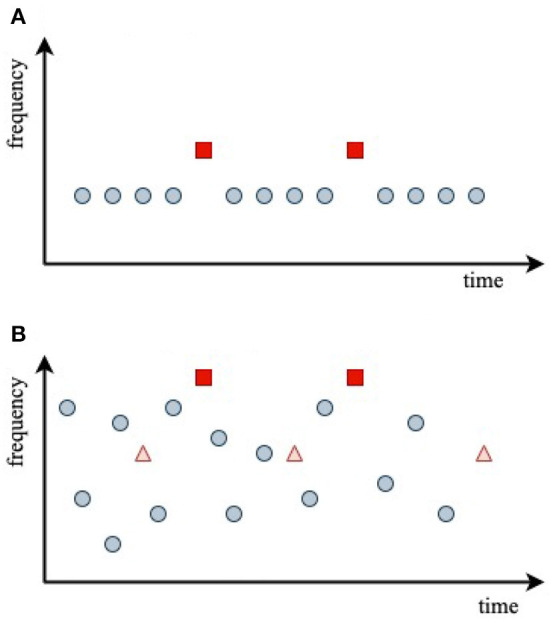
Stimulus sequences used in the experiments analyzed, with central stimulus frequencies indicated by dots at the stimulus onset times. **(A)** Oddball paradigm. **(B)** Random-frequency sequence with probe stimuli shown as triangles and squares.

We now apply the above theory to model ERs in two studies from the literature: (i) an oddball paradigm in which the frequency offset between standard and deviant stimuli is varied to examine how discriminability affects the MMN (Sams et al., 1985) and (ii) a series of fixed-frequency probe tones inserted into a random-frequency tone sequence, in which the ER has been shown to depend on the probability of background tones in the vicinity of the probe frequency (Garrido et al., 2013). These are illustrated in Figures 4A, B, respectively.

### 3.1 Oddball sequence

Figure 4A schematically shows the auditory oddball paradigm used by Sams et al. (1985) to investigate the effects of frequency discriminability on the difference between *D* and *S* responses. It is common to term the difference between the two responses the *mismatch negativity* (MMN), with


(41) $$\begin{array}{c} \text{MMN}\left(D,S,t\right)=D\left(t\right)-S\left(t\right). \end{array}$$

The MMN can be defined for any pair of responses, but it is most common to use the limiting form of *S*(*t*) after a long sequence of identical stimuli as the reference. We write this form as *S*_∞_(*t*).

In the experiments of Sams et al. (1985), a series of 1,000 Hz standard tones (S) of duration τ = 50 ms with ~1 ms rise and fall times was presented with an interstimulus interval of 1 s. These were replaced by deviant tones D with a probability of 0.2, which differed only in their frequency, which was fixed at 1,002, 1,004, 1,008, 1,016, or 1,032 Hz, respectively, in each of five trials.

At 1,000 Hz, Δ*f*_nat_ ≈ 3 Hz, while τ = 50 ms implies Δ*f*_*u*_ ≈ 20 Hz, so Δ*f* ≈ 20 Hz, dominated by the spectral width of the tone burst. Equations (24) and (25) then imply that ρ = 0.1, 0.2, 0.4, 0.8, 1.6 and ζ = 5 − 10. Hence, we predict that deviant frequencies of 1,002 and 1,004 Hz would produce *S*-like responses, those of 1,016 and 1,032 Hz would be *D*-like, and those of 1,008 Hz would be intermediate with significant *D*-like characteristics.

In this study, we denote the unadapted deviant response as $D_{1}$. Therefore, a baseline MMN_0_ can be defined as


(42) $$\begin{array}{c} {\text{MMN}}_{0}\left(t\right)=\text{MMN}\left(D_{1},S_{\infty },t\right)=D_{1}\left(t\right)-S_{\infty }\left(t\right). \end{array}$$

**Figure 5:**
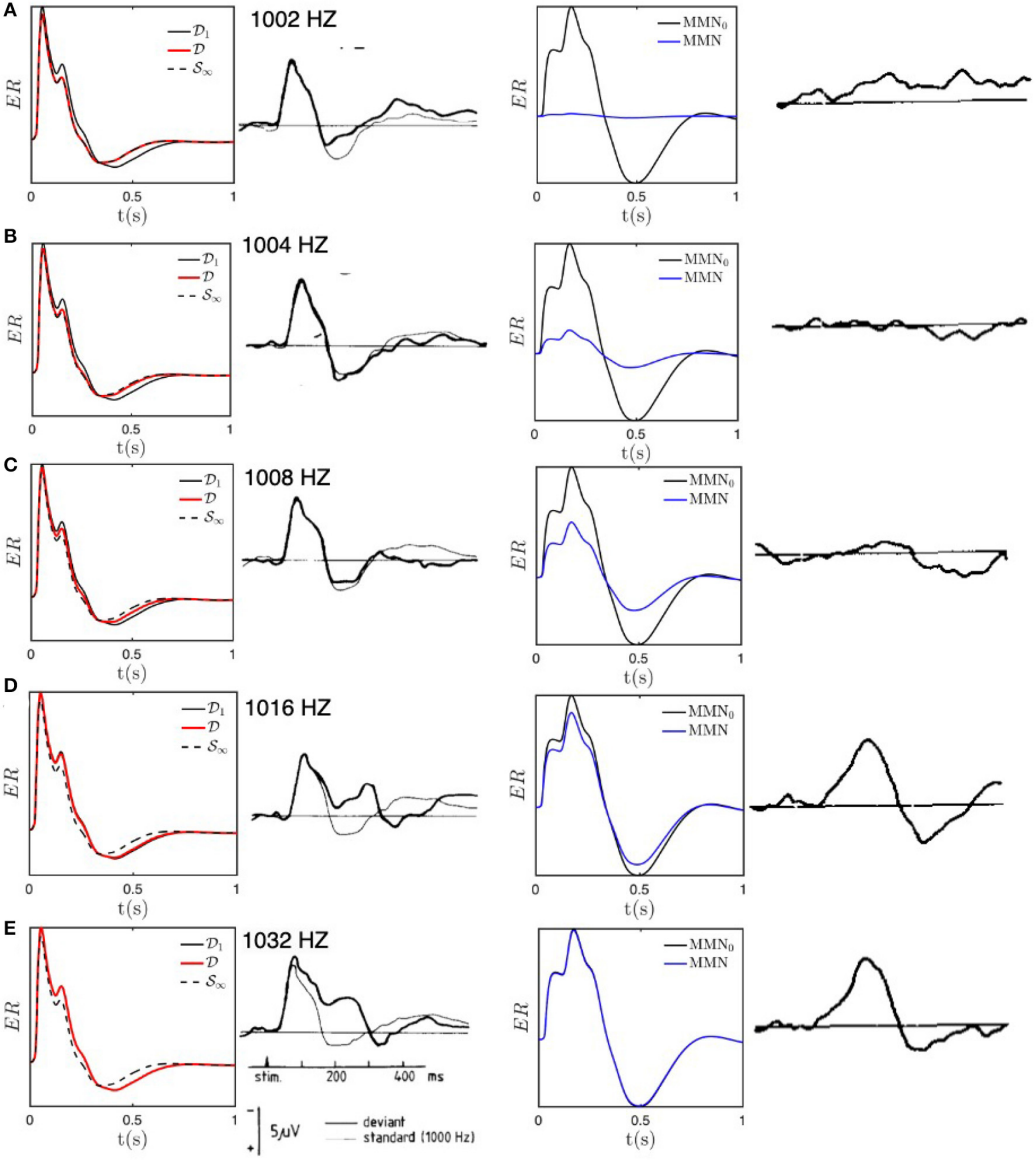
Model *S* and *D* responses for different deviant frequencies compared with experimental results adapted from Sams et al. (1985). Each row presents the results for a particular deviant frequency, as labeled in the second column. For each frequency, the first column shows model predictions for ERs excited with the deviant frequency (red) compared with the baseline *D*_1_ response (black solid) and the fully adapted *S*_∞_ response (black dashed); the second column shows the corresponding experimental result for deviant (heavy line) and standard (light line) stimuli; the third column shows the model MMN (solid) compared with MMN_0_ (dotted), and the final column shows the experimental MMN. **(A)** ERs and MMNs for deviant frequency *f*_*D*_ = 1, 002 Hz. **(B)** Same as **(A)** for *f*_*D*_ = 1, 004 Hz. **(C)** Same as **(A)** for *f*_*D*_ = 1, 008 Hz. **(D)** Same as **(A)** for *f*_*D*_ = 1, 016 Hz. **(E)** Same as **(A)** for *f*_*D*_ = 1, 032 Hz.

Figure 5 compares experimental results with the results of our numerical calculations for the above parameters; we use regularly spaced D stimuli, with one every 5 stimuli, to avoid the need to average. Figure 5A shows that the *D* responses for 1, 002 Hz are almost equal to the *S* response, with the *D* response slightly sharper and only a very small MMN. These findings are in agreement with experimental results by Sams et al. (1985) and are as expected because the 2 Hz frequency offset is much smaller than Δ*f* ≈ 20 Hz, so all stimuli cause adaptation in overlapping regions of the cortex. As the frequency offset increases through Figures 5B–E, the response progressively evolves away from *S*_∞_ toward *D*_1_, and is nearly identical to the latter for offsets of 16 and 32 Hz, with a correspondingly larger MMN that approximates MMN_0_. All these results are in accord with the experimental findings of Sams et al. (1985) and imply that our estimate of Δ*f* provides a good estimate of when the transition is complete; at half this value, an intermediate form is seen, as is evident in Figure 5C. Note that the residual differences between the theoretical and experimental curves cannot be considered to be significant because only six subjects' data were averaged to obtain these curves and ERs typically exhibit significant intersubject variation.

Individual ERs occur on timescales of a few hundred ms, which are much shorter than the adaptation timescale of 5–10 s. Hence, they can be viewed as being the impulse responses of a cortical region that has adapted from having its initial gains [_*G*_*ab*_]*i*_ (see Table 1) to gains determined by the slow adaptation parameters *h*_*ab*_, at the end of a long sequence of standard stimuli. In our case, we write the latter gains as [_*G*_*ab*_]*f*_ and state their values after 10 stimuli in Table 1. Comparison of the initial and final gains in Table 1 shows that the largest fractional changes within the corticothalamic system involve increases in the magnitudes of inhibitory gains (especially, *ei* and *sr*) and reductions in excitation (especially, *es* and *se*); there is a countervailing increase in the *sn* gain where stimuli enter the system. Overall, this is consistent with the overall level of activity being approximately maintained, but there being a substantially lower positive feedback in the corticothalamic loop that is comprised of the *es* and *se* connections. This loop is chiefly responsible for generating ~10 Hz alpha oscillations, so the reduction in its loop gain due to adaptation is consistent with the lower amplitude of such oscillations in the adapted (standard) response than in the initial (deviant) one. These results accord with our previous findings (Robinson et al., 2021), but with simultaneous and improved matches to typical standard and deviant responses.

### 3.2 Random-frequency sequence with probe stimuli

In the experiment of Garrido et al. (2013), illustrated in Figure 4B, subjects were presented with a random-frequency sequence of tones that could have either a narrow overall frequency distribution or a broad one. Superposed on this were two sequences of randomly spaced, fixed-frequency probe tones, one at the 500 Hz mean frequency of the random distribution, termed standard, S, and one at four times that frequency, termed deviant, D. Each probe sequence contained 10% of the overall number of stimuli. A key aim of the experiment was to explore how the *S* and *D* responses depended on the breadth of the background random frequency distribution and the frequency of the probe relative to its center—i.e., on the relative probability that random stimuli were in the vicinity of a given probe frequency.

Garrido et al. (2013) used stimuli with τ = 50 ms, rise and fall times of 10 ms (hence, an effective duration of 40 ms between half-maximum points), and interstimulus interval of 500 ms. The mean frequency of the Gaussian random distribution was 500 Hz, and it had a logarithmic standard deviation σ of either 0.5 octaves (narrow distribution, N) or 1.5 octaves (broad distribution, B). The probe frequencies were 500 and 2,000 Hz, and the timescale of their results implied *t*_*H*_ ≈ 10 s.

Garrido et al. (2013) published average responses, binned according to the quantity *N*_*a*_, which was the number of immediately preceding tones that all fell outside a frequency window of width Δ*x* of 1/3 octave (i.e., about 130 Hz each side of the 500 Hz probe tones, and 520 Hz each side of the 2,000 Hz probe tones). Large *N*_*a*_ was thus a very coarse-grained proxy for *not* being a recent stimulus at a nearby frequency, but the 1/3 octave range was not chosen based on the cortical response properties. We note that the experimental parameters give Δ*f* ≈ 20 Hz, which corresponds to spreads of only Δ*x* = 0.07 octaves at 500 Hz and 0.018 octaves at 2,000 Hz, so the bandwidth involved in calculating *N*_*a*_ is too wide for precise comparisons.

**Figure 6:**
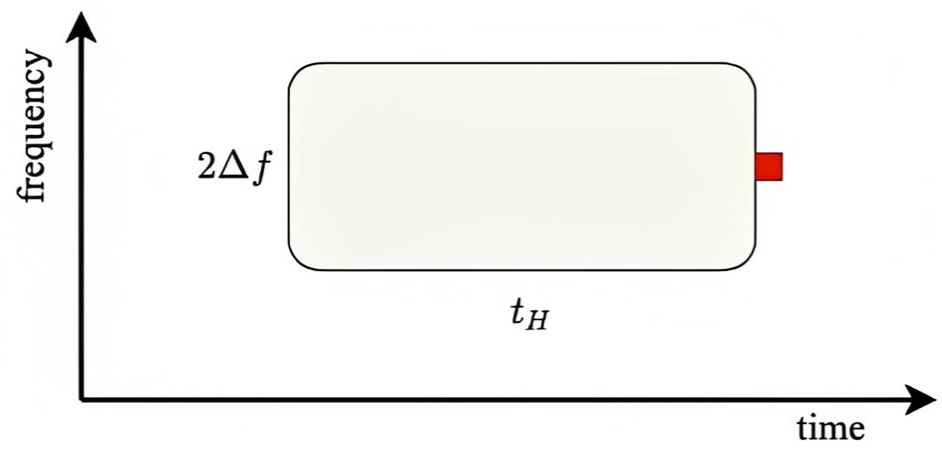
Schematics of adaptive window for a given stimulus of a particular frequency. The red square shows the arrival of a stimulus and the window determines the adaptive interval within which previous stimuli can have adaptive effects on the response. Similarly, the stimulus in question can affect responses in a similar window that follows it.

When considering a given probe frequency corresponding to *x*_*p*_, we can rewrite Equation (25), for the typical number of prior stimuli that affect a given response, as


(43) $$\begin{array}{ccc} \zeta & \approx & Rt_{H}\frac{2\Delta x}{\sigma \sqrt{2\pi }}\exp\left[-\frac{{\left(x_{p}-\bar{x}\right)}^{2}}{2\sigma^{2}}\right], \end{array}$$

where $\bar{x}$ corresponds to 500 Hz and 2Δ*x* appears because this is the total range around *x*_*p*_ that drives adaptation at *x*_*p*_. Using the above parameter values, we find ζ ≈ 2.3 for the SN condition (standard probe amid a narrow background distribution), ζ = 0.75 for the SB condition, ζ = 1.8 × 10^−4^ for the DN condition, and ζ = 0.075 for the DB condition. This implies that probe responses should be close to the fully adapted *S* form for the SN and SB conditions and close to the unadapted *D* form for the DB and DN conditions. Figure 6 illustrates the adaptation window that underlies Equation (43). The red square shows the arrival of a probe stimulus. Prior stimuli within a time *t*_*H*_ seconds before and within a frequency range of ±Δ*f* adaptively affect the ER to the probe, particularly if are recent and close in frequency. The more prior stimuli lie in the window, the closer the ER will be to a fully adapted standard *S*_∞_, whereas if the window is empty of prior stimuli, the ER will be close to the deviant *D*_1_.

The above values of ζ for the four conditions studied by Garrido et al. (2013) shows that the predictions are consistent with the experimental results: in the cases where ζ≪1, which correlates with large *N*_*a*_, Garrido et al. (2013) found D-like (unadapted) responses, whereas for ζ ≳ 1, the responses were more S-like, owing to greater adaptation. These results thus accord with our expectation that there should be little adaptation if there have been few or no stimuli within the window shown in Figure 6. However, the 1/3 octave bandwidth used in defining *N*_*a*_ is much larger than the physical width, so the correlation is weakened because many data points with moderate *N*_*a*_ do not involve significant adaptation, but were included along with strongly adapted cases in the experimental averages.

The above results suggest a more efficient use of data, and a streamlined experimental procedure, in ER experiments on random frequency stimuli. Instead of using separate fixed-frequency probes of the responses to random stimuli, the random stimuli can be used to probe one another. In this case, the responses would simply be binned and averaged according to the value of ζ, and *N*_*a*_ would not be used; one could even use the actual value of the number of stimuli in the window shown in Figure 6, with a weight function to smooth the edges of the window. Moreover, to improve statistics toward the edges of the frequency distribution, a uniform distribution in frequency or its logarithm, rather than a Gaussian, could be employed.

## 4 Summary and conclusion

In this study, we have generalized our previous theory of evoked responses with adaptation to allow for frequency-dependent responses, to obtain criteria for when significant adaptation occurs, and to determine whether adaptation suffices to reproduce standard (adapted), deviant (unadapted), and intermediate responses. The results have been applied to explain the response dynamics seen in experiments in which standard and deviant tones differ only in frequency, and in which random-frequency tones are presented. The main results are as follows:

- Extension to frequency-dependent adaptation was achieved by allowing for the intrinsic spread in frequency of a tone burst due to its finite duration plus the known spread due to the divergence in projections to the auditory cortex via the tonotopic map. These effects mean that adaptation at the nominal tonotopic location of a given frequency is affected by stimuli at nearby frequencies.
- Stimuli cause adaptation at neighboring frequencies and subsequent times, causing later stimuli in the affected zone to produce evoked responses more like standards than deviants. The quantities ρ and ζ defined in Equations (24) and (25) can be used to quantify the affected frequency–time range: when numerous stimuli are received, significant adaptation occurs for ρ ≲ 1 and ζ ≳ 1. Typically, these correspond to adaptive effects from one stimulus affecting the responses to other stimuli within a few Hz and ~5 s.
- The main gain changes tended to increase cortical inhibition and reduce positive corticothalamic feedback, while maintaining overall mean brain activity levels by increasing the gain where external stimuli enter the thalamus. However, positive feedback via the corticothalamic loop was significantly reduced, leading to lower amplitude ~10 Hz oscillations in the adapted (standard) response than in the initial (deviant) one. These results were consistent with our previous study (Robinson et al., 2021). Good matches to both standard and deviant responses, and during adaptation driven by a sequence of stimuli, were obtained using a single set of parameters.
- The results were found to be consistent with experimental results for oddball sequences in which the deviant stimuli differed only in their frequency relative to the standards (Sams et al., 1985).
- In the case of random-frequency stimulation (Garrido et al., 2013), the criteria mentioned in (ii) were found to be consistent with the experimental results. Specifically, significant adaptation occurs if expected number of stimuli within the adaptation time–frequency window exceeds about 1, as expressed by Equation (43). By using the present criteria and binning according to the number of prior stimuli in the window shown in Figure 6, every stimulus can be used as a probe of the adaptive effects due to prior stimuli at nearby frequencies and times, rather than having to rely on probe stimuli at specific frequencies. This approach would make fuller use of such data, thereby enabling shorter experimental protocols.

Overall, these results significantly extend the range of experiments on evoked response sequences that can be explained by adaptive effects in sensory cortex within a neural field theory framework, showing that many mismatch negativity findings can be explained by adaptation at relevant points in the tonotopic map, so long as adaptation exists and notwithstanding some debate as to its exact mechanisms (Ruusuvirta, 2021). Future study could usefully apply similar methods to investigate deviant stimuli that differ only in intensity or duration, sequences of descending tones in which one tone is repeated, or more abstract deviance rules. Such analyses will help to distinguish local adaptive effects from those of top-down feedbacks from higher cortical areas—an essential contribution toward probing the levels at which different aspects of stimuli are processed.

## Data availability statement

Information for existing publicly accessible datasets is contained within the article.

## Author contributions

TB-J: Conceptualization, Formal analysis, Investigation, Methodology, Software, Visualization, Writing - original draft, Writing - review & editing. NG: Formal analysis, Investigation, Methodology, Software, Visualization, Writing - original draft, Writing - review & editing. AM: Formal analysis, Investigation, Methodology, Software, Writing - original draft, Writing - review & editing. PR: Conceptualization, Formal analysis, Funding acquisition, Investigation, Methodology, Project administration, Resources, Supervision, Writing - original draft, Writing - review & editing.
